# A Quantitative Indicator-Based Framework for Sustainability-Driven Selection of Conventional and Smart Materials in the Built Environment

**DOI:** 10.3390/ma19173712

**Published:** 2026-08-31

**Authors:** Paolo Trucillo, Fatah Fatehi Peikani

**Affiliations:** 1 Dipartimento di Ingegneria Chimica, dei Materiali de della Produzione Industriale, University of Naples Federico II, Piazzale Vincenzo Tecchio, 80, 80125 Naples, Italy; 2 Department of Structures for Engineering and Architecture, University of Naples Federico II, Via Forno Vecchio, 36, 80134 Naples, Italy; fatah.1989@gmail.com

**Keywords:** sustainability: indicators, smart materials, material selection, urban furniture, built environment, shape memory materials

## Abstract

The transition towards sustainable cities requires material selection strategies capable of balancing environmental, economic and social performance while accounting for the emerging functionalities offered by smart materials. This work proposes a sustainability-driven methodology based on normalized multi-dimensional indicators for the objective comparison of functionally equivalent conventional and smart materials in the built environment and demonstrates its application through the parametric design of an urban bench. The quantitative assessment showed substantial differences among the investigated materials: Shape Memory Polymers (SMPs) resulted in a panel mass of 5 kg, CO_2_ emissions of 2.4 kg CO_2_/kg, and embodied energy of 37 MJ/kg, compared with 32 kg, 13 kg CO_2_/kg, and 260 MJ/kg for NiTi, and 40 kg, 15 kg CO_2_/kg, and 320 MJ/kg for magnetic Shape Memory Alloys, respectively. Finite element analysis further demonstrated the influence of structural design, with the maximum principal stress decreasing from approximately 0.073 MPa at a panel thickness of 10 mm to 0.015 MPa at 50 mm, corresponding to a reduction of approximately 79%. The results demonstrate that smart materials do not inherently represent more sustainable alternatives than conventional materials; rather, their adoption should be justified when their adaptive functionalities provide measurable benefits capable of compensating for their environmental and economic burdens. The proposed framework provides a practical decision-support tool that shifts material selection from property-driven choices toward function-oriented, sustainability-based design, supporting more informed decisions for next-generation urban infrastructure.

## 1. Introduction

Smart materials have attracted increasing attention in the built environment due to their ability to adapt to external stimuli, improving building energy efficiency, functionality, and resilience [1]. Assessing their contribution to sustainable construction requires robust methodologies integrating environmental, economic, and social sustainability indicators [2]. However, the effective implementation of these frameworks remains challenging because of the complexity of weighting sustainability criteria, incorporating resilience metrics, and adapting existing certification systems to emerging smart materials. Consequently, further research is required to develop robust methodologies for assessing the sustainability performance of smart materials in the built environment.

The growing demand for sustainable buildings is driven by the increasing environmental impact of the construction sector, which is responsible for a substantial share of global energy consumption, greenhouse gas emissions, and raw material use [3]. Consequently, sustainable development has become a fundamental objective of the built environment, extending beyond environmental concerns to encompass economic and social dimensions [4]. This transition has also promoted the adoption of circular construction principles, resource-efficient materials, and resilience-oriented design strategies, highlighting the need for reliable methodologies capable of evaluating sustainability in an integrated and quantitative manner [5].

To address these challenges, the concept of sustainability has progressively evolved from resource conservation to a broader and more integrated framework [6]. Today, sustainability is widely recognized as a dynamic process that combines environmental, economic, and social dimensions while promoting resilience, circular resource management, and long-term value creation [7]. This multidimensional perspective highlights the need for comprehensive assessment methodologies capable of simultaneously evaluating the different aspects of sustainability in the built environment.

The sustainability framework has been further expanded by recognizing that sustainable development should not only balance environmental, economic, and social dimensions but also ensure environmental protection, equitable resource distribution and the well-being of both present and future generations [8]. Within this framework, Sustainability Indicators (SI) have emerged as essential tools for quantitatively evaluating the environmental, economic, and social performance of construction materials and technologies [9]. Several assessment frameworks have been proposed to support decision-making in sustainable construction, integrating life-cycle analysis, resilience, and stakeholder perspectives [10,11]. Nevertheless, current methodologies still present significant limitations, particularly in the evaluation of emerging smart materials, due to the complexity of combining multiple sustainability dimensions into reliable and practical assessment methods [12]. Consequently, further research is needed to develop comprehensive and adaptable indicator-based approaches capable of supporting the selection and design of next-generation materials for the built environment [13].

Despite these advances, selecting materials for the built environment remains a complex multi-criteria decision-making problem. Construction materials must simultaneously satisfy technical, environmental, economic, and social requirements, which are often conflicting [14]. For example, a material with superior mechanical or thermal performance may exhibit a higher environmental footprint or economic cost, whereas a more sustainable alternative may compromise durability or functionality. As a result, material selection can no longer rely on a single performance criterion but requires integrated evaluation methods capable of balancing multiple sustainability dimensions.

Indeed, existing methodologies generally evaluate sustainability dimensions separately and provide limited guidance for integrating multiple indicators into a practical decision-support framework for smart material selection [15]. This limitation becomes even more relevant for emerging smart materials, whose multifunctional behavior cannot be adequately captured through conventional assessment approaches. Therefore, there is still a need for robust and adaptable methodologies capable of quantitatively comparing conventional and smart materials using a coherent set of sustainability indicators.

The present approach builds upon previous work by the authors on sustainability-oriented material selection for furniture applications [16], while introducing a substantially different methodological scope. While the previous study primarily combined environmental assessment and structural optimisation for the selection of conventional materials, the present study is specifically designed to compare conventional and smart materials on the basis of functional equivalence. Environmental, economic, mechanical and safety-related social indicators are integrated into a common normalized assessment to determine whether the additional functionality provided by a smart material can justify its associated sustainability burdens. Therefore, the proposed methodology shifts the decision process from property-based material ranking toward function-oriented sustainability assessment.

## 2. From Methodology to Real Urban Needs

Although the proposed methodology is general and applicable to different smart materials, its practical relevance becomes evident when addressing real challenges in urban environments. Many public spaces still rely on conventional urban furniture that no longer satisfies contemporary requirements in terms of comfort, durability, accessibility, adaptability, and sustainability.

**Figure 1:**
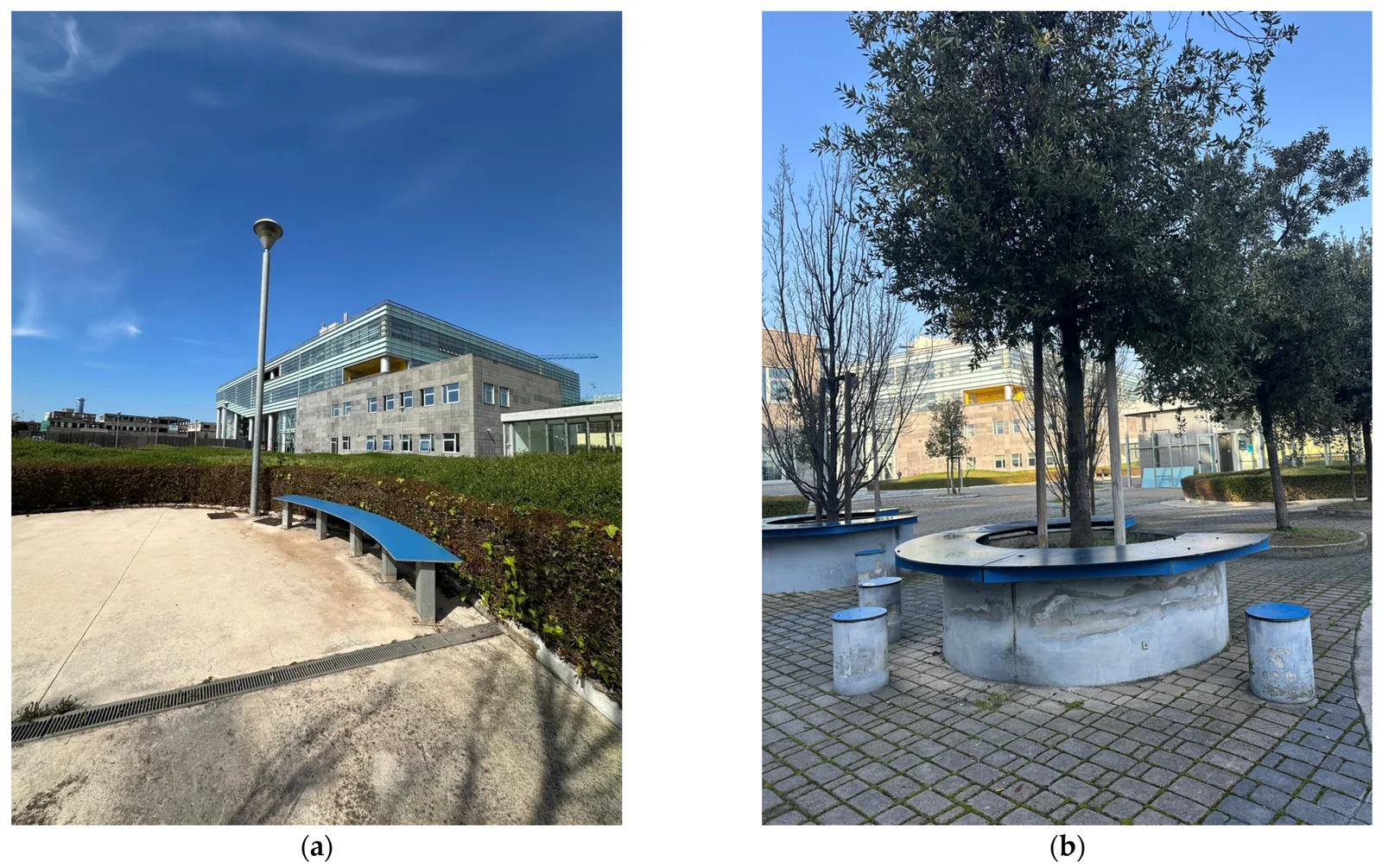

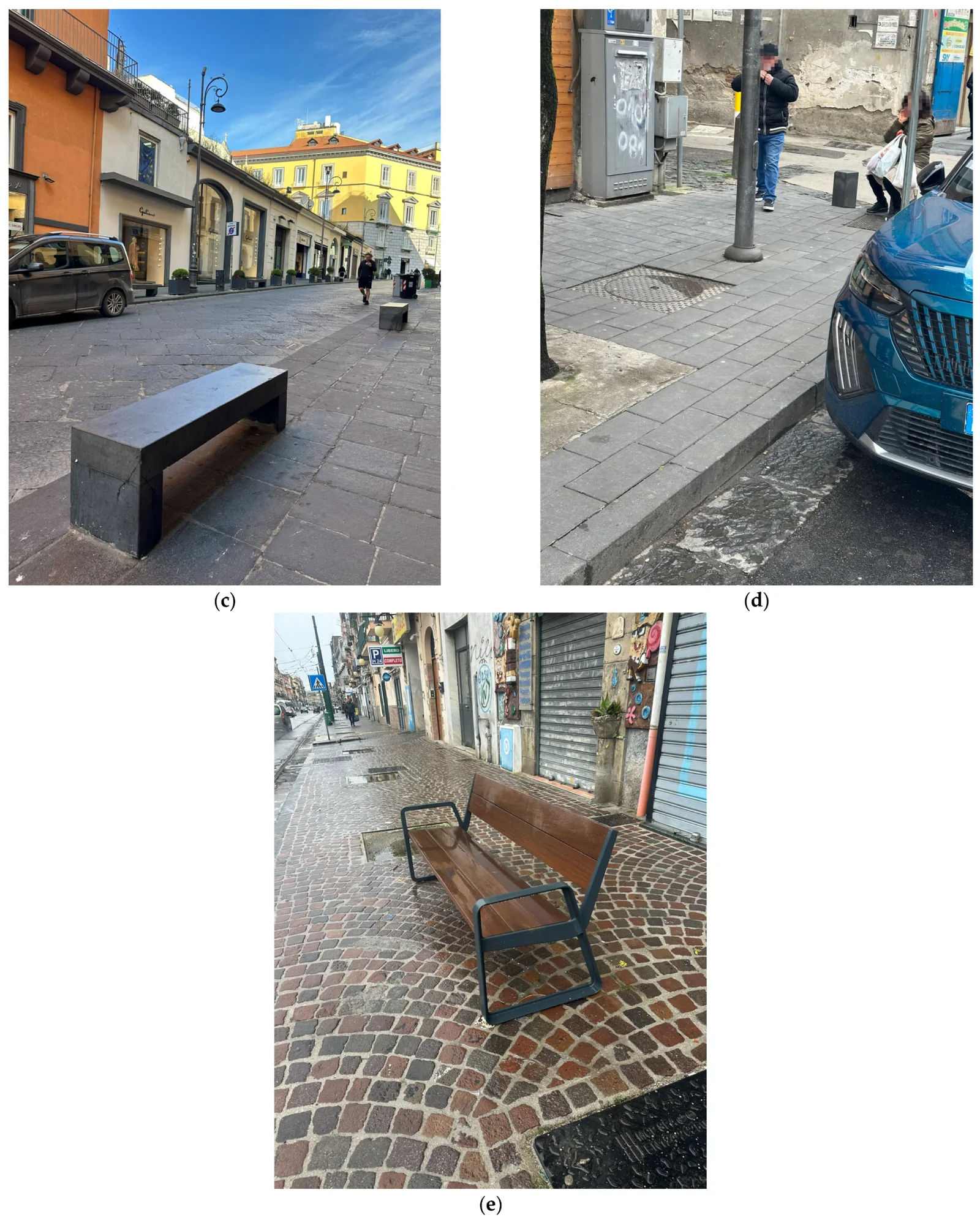
Representative examples of existing urban furniture in Naples, Italy: (**a**) seating area within the San Giovanni a Teduccio university campus; (**b**) student seating area within the San Giovanni a Teduccio university campus; (**c**) urban bench in Piazza Amedeo; (**d**) existing seating solution in Corso Secondigliano; (**e**) conventional urban bench in San Giovanni a Teduccio. All photographs were taken by the authors.

The southern Italian city of Naples represents a meaningful example of this scenario (Figure 1). Figure 1a shows a conventional seating area within the San Giovanni a Teduccio university campus, while Figure 1b illustrates an additional student seating area within the same campus. Figure 1c shows an example of conventional urban furniture in Piazza Amedeo, whereas Figure 1d illustrates an existing seating solution in Corso Secondigliano. Finally, Figure 1e provides an additional example of a conventional urban bench in San Giovanni a Teduccio. These examples show different contexts in which existing urban furniture could benefit from material, functional or ergonomic redesign. Urban regeneration therefore offers an opportunity not only to replace or redesign existing furniture but also to reconsider the materials adopted for future installations. Within this context, the proposed methodology provides designers and public administrations with a quantitative decision-support framework capable of comparing alternative material solutions through objective sustainability indicators rather than subjective preferences or economic considerations alone. For these reasons, smart materials should not be regarded as universal replacements for conventional construction materials. Instead, their use should be justified only when their adaptive behavior provides measurable functional benefits that cannot be achieved through traditional solutions. Materials such as shape memory alloys and shape memory polymers introduce unique capabilities, including reversible deformation, adaptive response, and multifunctionality; however, these advantages are generally accompanied by higher manufacturing costs and greater environmental burdens. Consequently, the selection of smart materials should be driven by clearly identified engineering requirements rather than by technological novelty alone [17,18,19].

An important outcome of this study is that the sustainability ranking of materials depends strongly on the engineering function under consideration. The comparison performed between functionally equivalent solutions shows that conventional materials remain preferable for applications where structural performance, durability, and low environmental impact are the dominant design requirements. Conversely, smart materials become competitive only in applications requiring adaptability, active response, or multifunctionality. This confirms that sustainability-driven material selection cannot rely on the intrinsic properties of a material alone but must consider the interaction between material behavior, functional requirements, and application context.

The use of normalized indicators [20] will allow materials characterized by different physical properties and units of measurement to be compared on a common basis, while radar diagrams facilitate the visualization of strengths, weaknesses, and trade-offs. Such an approach can support engineers, architects, and urban planners during the early design stages, when material selection has the greatest influence on the overall sustainability of the project.

## 3. Methods

### 3.1. Sustainability Assessment Framework

The sustainability assessment was performed using a quantitative multi-criteria tool integrating environmental, economic and social indicators. Such an approach is particularly suitable for the built environment, where the sustainability of urban furniture and construction materials depends on multiple and often conflicting criteria. Multi-criteria decision-making approaches provide an effective means of comparing different materials by integrating heterogeneous indicators into a common evaluation framework. As also reported in the literature [16,21,22], to enable direct comparison among indicators characterized by different units and scales, all values were normalized using Equation (1):

(1) $$\mathrm{Score},\;\%\;=\;\frac{\mathrm{Actual}-\mathrm{Worst}}{\mathrm{Best}-\mathrm{Worst}}\times 100$$

where a score of 0% represents the worst sustainability performance and a score of 100% represents the best sustainability performance for each indicator. The Actual value is the numeric information that we want to evaluate and compare with other simulations, materials or with previous registered performances. The normalized indicators were subsequently represented using radar diagrams, providing an intuitive visualization of the sustainability profile of each material and facilitating the identification of strengths, weaknesses, and trade-offs among the considered criteria.

Sustainability indicators were derived from currently available literature and representative datasets, while several emerging smart materials still lack consolidated long-term environmental and durability data. Furthermore, the structural analysis was performed on a single parametric bench geometry, and therefore the quantitative results should not be generalized to every possible urban furniture configuration. Future research should extend the proposed methodology to additional smart materials, different structural typologies, and complete life-cycle assessments, while also incorporating experimental validation and long-term field performance [23,24,25].

### 3.2. Definition of Specific Indicators

Since smart materials exhibit multifunctional behavior, their sustainability cannot be assessed solely in terms of technical performance. A comprehensive evaluation requires simultaneous consideration of environmental, economic and social aspects throughout the material life cycle [26]. Accordingly, the sustainability assessment was based on a set of indicators selected from the literature according to their relevance to the built environment and their applicability to both conventional and smart materials.

The selected indicators consider resource consumption, environmental impacts, technical and economic performance, as well as social implications, providing a more general and wider scenario for material comparison [27,28]. Environmental indicators evaluate the efficient use of resources, recyclability, waste generation, toxicity, and greenhouse gas emissions. Economic indicators assess operational efficiency, durability, maintenance requirements, cost-effectiveness, and market readiness, while social indicators account for occupational safety, employment generation, community benefits and social acceptance. Together, these indicators provide a comprehensive basis for quantitatively comparing alternative materials and supporting evidence-based material selection for sustainable construction.

The selected indicators were specifically chosen to show the multifunctional nature of smart materials. Unlike conventional construction materials, smart materials are capable of dynamically adapting their physical or chemical properties in response to external stimuli, such as temperature, humidity, pressure, or electromagnetic fields. Consequently, their sustainability assessment cannot rely exclusively on traditional environmental indicators but must also account for functional performance, durability, resource efficiency, adaptability and long-term socio-economic benefits. The proposed methodology combines conventional sustainability metrics with indicators capable of reflecting the additional value provided by adaptive materials, ensuring a comprehensive and balanced comparison between conventional and smart solutions in the built environment. The indicator set presented in Table 1 was subsequently applied to representative case study involving both conventional and smart materials, allowing a quantitative comparison of their sustainability performance through the normalization procedure described in Section 3.1.

For the social dimension, job creation (JC), community benefits (CB), and social acceptance (SA) were included in the quantitative assessment using a semi-quantitative five-point scale, ranging from 1 (least favourable condition) to 5 (most favourable condition). This approach was adopted because, unlike environmental or mechanical properties, these indicators cannot generally be expressed as intrinsic material properties and depend on factors such as supply-chain maturity, employment intensity, local economic interactions, technological maturity and stakeholder perception.

Job creation was evaluated considering the employment-generation potential associated with material production, processing, installation, and maintenance; community benefits considered the potential contribution to local employment, skills development, circular-economy activities, and local supply chains; and social acceptance accounted for technological maturity, familiarity of the material in built-environment applications, market confidence, and potential implementation barriers.

The resulting values were subsequently normalized according to Equation (1), using 1 and 5 as the Worst and Best reference values, respectively. These scores should therefore be interpreted as comparative decision-support indicators rather than intrinsic social properties of the materials.

Adaptability was considered as a functional material attribute rather than as a sustainability indicator itself. For shape-memory materials, this functionality can be quantitatively described through material-specific parameters such as recoverable strain, actuation strain, recovery ratio, and transformation or activation temperature. These parameters provide a measurable description of the capacity of a material to respond to an external stimulus and recover or modify its configuration. Therefore, adaptability should not be interpreted here as a qualitative sustainability score, but as a measurable functional requirement that determines whether the additional environmental, economic, and social burdens associated with a smart material can be justified for a specific application.

It is worth noting that transition towards sustainable construction requires decision-making processes that consider not only the functional performance of materials but also their environmental, economic, and social impacts throughout their life cycle. While conventional construction materials have been extensively characterized in terms of cost, mechanical performance, and durability, smart materials introduce additional functionalities, including adaptability, self-healing capability, energy harvesting, and environmental responsiveness. These features can significantly improve the operational performance of buildings but may also involve higher production costs, increased embodied energy, or more complex manufacturing processes. Consequently, the selection of materials for the built environment should not rely exclusively on technical performance or individual sustainability metrics. Instead, it requires am overall evaluation tool capable of balancing the benefits provided by smart functionalities against their associated environmental, economic, and social impacts.

### 3.3. Presentation of Case Study

#### 3.3.1. General Considerations

Rather than comparing materials based solely on their intrinsic properties, the proposed approach evaluates conventional and smart solutions performing the same engineering function. In the preliminary sustainability screening, an identical reference geometry was adopted for all materials to isolate the effect of material selection. However, identical geometry alone does not imply full structural equivalence, since materials differ in strength and stiffness. Therefore, functional equivalence should also require verification of common structural and serviceability requirements through the subsequent structural analysis and, where necessary, adjustment of the relevant design parameters, such as panel thickness. This two-step approach enables sustainability performance to be compared under a common reference configuration while ensuring that the final material solution remains consistent with the required engineering function [29].

Smart materials are increasingly being integrated into urban furniture, transforming conventional passive structures into multifunctional systems capable of interacting with both users and the surrounding environment. Besides providing structural support, these materials can integrate sensing, adaptive lighting, energy harvesting, environmental monitoring, and shape-changing functionalities, significantly enhancing both sustainability and user experience.

A representative example is the transparent shape-editable wood composite (P-SEFTW), developed through the incorporation of fluorescent dyes and epoxy vitrimers into delignified wood. This material combines transparency, programmable shape memory, and light-guiding capabilities while maintaining the renewable nature of its wooden substrate. These characteristics make it particularly suitable for adaptive urban furniture, extending product lifetime while reducing material consumption and maintenance requirements [30].

Another promising class of smart materials is represented by MXene-based composites. Owing to their high electrical conductivity, mechanical flexibility, and chemical stability, MXenes enable multifunctional urban furniture capable of energy harvesting, environmental sensing, air-quality monitoring, electromagnetic shielding, and Internet of Things (IoT) connectivity. These features transform conventional urban furniture into active components of smart city infrastructure while improving durability and resource efficiency.

Compared with traditional materials, these smart solutions provide longer service life, lower maintenance requirements, improved adaptability, and additional environmental services, supporting the transition toward more sustainable and resilient urban environments. Although smart materials generally involve higher initial investment costs than conventional alternatives, several studies have demonstrated that their improved durability, reduced maintenance requirements, enhanced operational efficiency, and longer service life frequently compensate for the higher acquisition costs throughout the product life cycle [31]. Additional economic benefits arise from circular economy strategies, including material recycling, CO_2_ reutilization, modular construction, and the integration of renewable energy systems. Nevertheless, large-scale implementation is still constrained by production scalability, supply chain limitations, market readiness, and the availability of specialized manufacturing technologies.

Beyond economic considerations, smart materials can also generate significant social benefits by improving accessibility through adaptive geometries, enhancing public safety via autonomous systems, monitoring environmental quality with embedded sensors, and contributing to pollutant reduction through advanced functional materials. These capabilities improve urban livability and citizens’ well-being, although their widespread adoption is still limited by regulatory, technical, economic, and social barriers that require multidisciplinary collaboration, technological standardization, and supportive policy measures.

In this context, smart materials are capable of mechanical adaptation and climate-responsive behavior, with particular emphasis on active shape-changing materials such as shape memory alloys (SMAs), selected for their reversible deformation, structural adaptability, and resilience under varying environmental conditions. Passive adaptive materials, such as humidity-responsive polymers and hydrogels, are considered only as complementary examples, whereas materials primarily based on optical, luminescent, or surface-adhesion mechanisms are excluded. This selection enables a consistent comparison with conventional materials performing equivalent engineering functions through the sustainability indicator framework previously described, with the aim of identifying the conditions under which smart materials provide measurable environmental, economic, and social advantages for applications in the built environment.

#### 3.3.2. Finite Element Method Analysis

The finite element analysis was introduced as a supporting design tool within the proposed sustainability-driven material-selection framework rather than as a stand-alone high-fidelity structural investigation. Its primary purpose was to provide a consistent mechanical comparison under common geometrical and loading conditions and to investigate the influence of panel thickness on the structural response. Accordingly, the numerical model was intentionally maintained at a level of complexity appropriate for comparative material screening.

**Figure 2:**
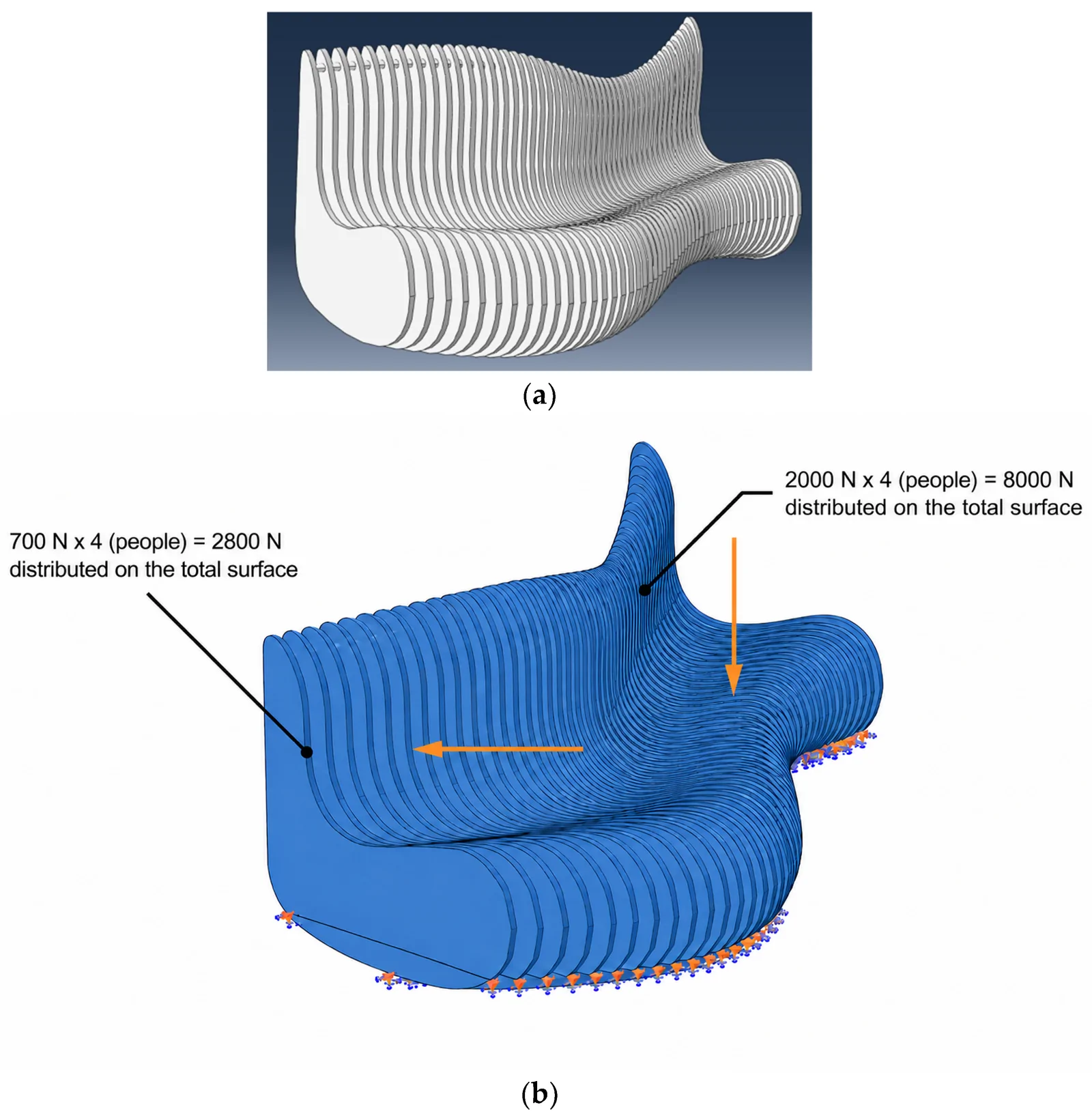
(**a**) Parametric bench geometry generated in Rhinoceros; (**b**) finite element model in Abaqus showing the applied loads and boundary conditions. Applied pressures directions are indicated with orange arrows.

The bench was generated as a fully parametric system using Rhinoceros (Robert McNeel & Associates (TLM, Inc.), Seattle, WA, USA), where the overall surface was first defined as a smooth continuous spline responding to ergonomic seating requirements for four simultaneous users. This base geometry was then discretized into 48 vertical rib-like panels, as shown in Figure 2a. The finite element model was developed in Abaqus (version Abaqus/CAE 2021 with the Abaqus/Standard solver, Dassault Systèmes SIMULIA Corp., Paris, France) using three-dimensional second-order tetrahedral solid elements, selected because of the complex and organically curved geometry of the bench. An automatic meshing procedure was adopted, with finer discretization in regions characterized by higher curvature and expected stress gradients. The same meshing strategy and element formulation were maintained for all investigated configurations to ensure that differences in the numerical response resulted from variations in material properties and panel thickness rather than from changes in the discretization procedure.

For the purposes of the comparative analysis, the materials were represented through simplified homogeneous linear-elastic models using the reference elastic properties and densities summarized in Table 2. This assumption enables a consistent comparison among materials characterized by substantially different mechanical behaviour. The adopted Young’s modulus, Poisson’s ratio, and density represent reference values appropriate for comparative modelling; however, material-specific phenomena such as the phase transformation of shape-memory alloys, polymer viscoelasticity, wood orthotropy, concrete cracking, plasticity and residual stresses were not explicitly modelled. Such phenomena would require dedicated constitutive models and material-specific experimental calibration and are beyond the scope of the present sustainability-oriented comparative study.

The loading conditions were defined according to Furniture Seating reference [32] methods for the determination of strength and durability, consistently with the approach adopted in our previous study [16]. In particular, the standard provides a seat static load of 2000 N and a backrest static load of 700 N for the corresponding seat-and-back loading condition.

Considering four users, resultant forces of 8000 N on the seating region and 2800 N on the backrest region were therefore considered in the present model. These loads were implemented as uniformly distributed pressures over the corresponding seat and backrest surfaces, with pressure magnitudes calculated to produce the prescribed resultant forces, rather than as concentrated forces or moments.

The seat and backrest loading conditions were applied simultaneously, thereby reproducing the combined loading condition considered in the reference testing procedure. The same loading configuration was consistently applied to all investigated material and thickness configurations. The applied distributed pressures and boundary conditions are illustrated in Figure 2b.

A formal mesh-convergence study was not performed because the FE analysis was intended to support comparative material and geometrical screening rather than to provide a high-fidelity structural validation or certification of the bench design. The same discretization strategy was therefore consistently applied to all configurations, and the numerical results were used primarily to identify comparative trends associated with material selection and panel thickness rather than to establish mesh-independent local stress or displacement values. This represents a limitation of the present numerical analysis; consequently, dedicated mesh-sensitivity, nonlinear constitutive modelling, and experimental structural validation would be required before translating the proposed conceptual design into a real engineering application.

## 4. Results

### 4.1. Sustainability Assessment of Conventional and Smart Materials

The most representative proposed materials were evaluated using the same bench geometry, composed of 48 panels with fixed areas and volumes. By keeping the geometry constant, the analysis isolates the effect of material choice on sustainability performance. Differences observed in the results are therefore linked to material properties rather than design variations. The selected indicators were quantified from literature data and engineering databases for all the investigated materials [33,34,35]. Since the indicators are expressed in different units and represent different sustainability dimensions, the raw values are first presented in Table 3 and subsequently converted into normalized scores according to Equation (1). This two-step procedure preserves data transparency while enabling an objective multi-criteria comparison.

**Figure 3:**
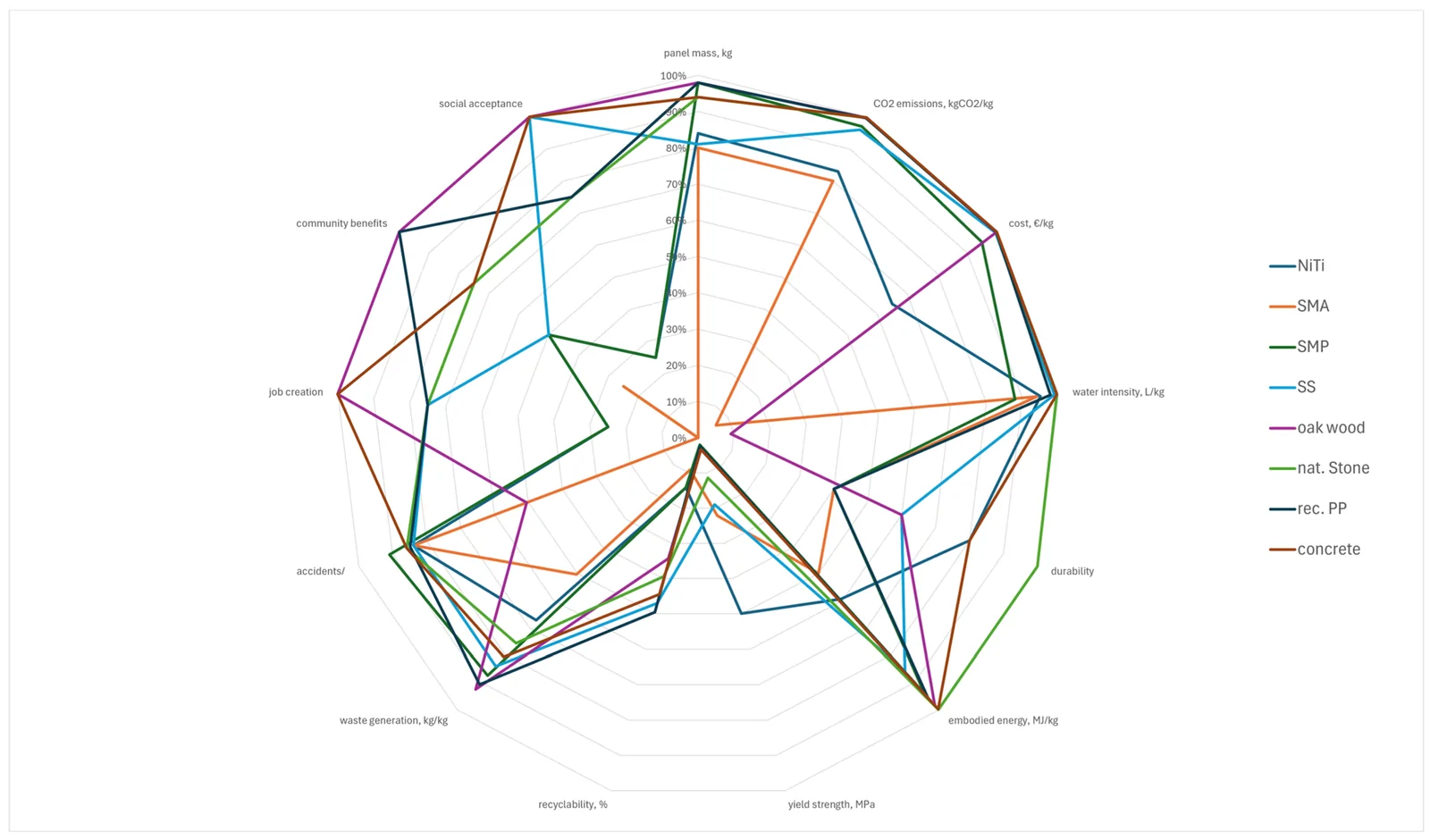
Normalized sustainability profiles of conventional and smart materials obtained through the proposed indicator-based methodology. The radar diagram integrates environmental, economic, mechanical, safety and social indicators, including job creation, community benefits, and social acceptance, supporting a multidimensional and function-oriented material selection.

Therefore, all indicators were normalized according to Equation (1) and represented using the radar diagram of Figure 3, allowing the simultaneous visualization of environmental, economic, mechanical, safety, and broader social performances. In particular, the inclusion of job creation, community benefits, and social acceptance extends the comparison beyond conventional environmental and technical criteria and provides a more comprehensive representation of the social dimension of material sustainability.

Figure 3 immediately shows that no material simultaneously maximizes all sustainability indicators. Instead, each material is characterized by a specific sustainability profile resulting from different trade-offs among environmental, economic, mechanical, and safety performances. This confirms the necessity of a multi-criteria assessment methodology, since conclusions based on a single indicator would inevitably lead to biased material selection. Unlike conventional comparison tables, the proposed radar representation enables the simultaneous visualization of heterogeneous sustainability indicators, providing an intuitive decision-support tool for identifying material-specific strengths and weaknesses.

Traditional materials exhibit a more balanced sustainability profile, combining low cost, reduced environmental impact, established durability, and high recyclability. Smart materials, conversely, excel only in specific functional dimensions associated with adaptive behavior, but generally require higher embodied energy, manufacturing costs, and environmental resources. Among them, Magnetic Shape Memory Alloys display the least favorable overall sustainability profile, whereas Shape Memory Polymers represent a more balanced compromise, combining lightweight characteristics with adaptive functionality and moderate environmental impacts.

The inclusion of the three additional social indicators further differentiates conventional and smart materials. Established materials such as oak wood, stainless steel, concrete, and natural stone generally achieve higher social scores, reflecting mature supply chains, greater employment-generation potential, and widespread familiarity in built-environment applications. Recycled polypropylene also performs favourably, particularly in terms of community benefits, owing to the potential involvement of local collection, sorting, recycling, and circular-economy activities. Conversely, NiTi, magnetic SMAs, and SMPs exhibit lower social scores, mainly because their production chains are more specialized and their adoption in the built environment remains comparatively limited. Among the smart materials considered, SMPs nevertheless retain a comparatively more balanced overall profile when environmental, economic, and social dimensions are considered together.

These findings demonstrate that smart materials should not be regarded as universal replacements for conventional materials. Rather, their adoption should be justified only when their adaptive capabilities provide measurable functional benefits that compensate for their additional environmental and economic burdens. Consequently, sustainability-driven material selection should focus on identifying the most appropriate material for a specific engineering function instead of seeking the universally “best” material.

### 4.2. Design of a Parametric Bench

As clarified in Section 3.3.2, the numerical analysis was not intended to reproduce the complete constitutive behaviour of smart materials or to provide a detailed structural optimisation of the bench. Instead, it was used to determine whether the adoption of a smart material such as NiTi, within the same reference design, leads to substantially different stress distributions compared with a conventional structural material such as stainless steel.

Accordingly, NiTi was represented using a simplified homogeneous linear-elastic material model. Phase transformation, superelasticity, and shape-memory effects were not activated in the present simulations. This simplification intentionally isolates the conventional elastic structural response of the material and allows a consistent comparison with stainless steel under identical geometry and loading conditions. Consequently, the numerical results presented here should not be interpreted as a prediction of the complete functional response of NiTi. In a real adaptive application, activation of phase transformation and superelastic or shape-memory behaviour could modify the local stress–strain response and would require a dedicated nonlinear constitutive model and material-specific experimental calibration.

**Figure 4:**
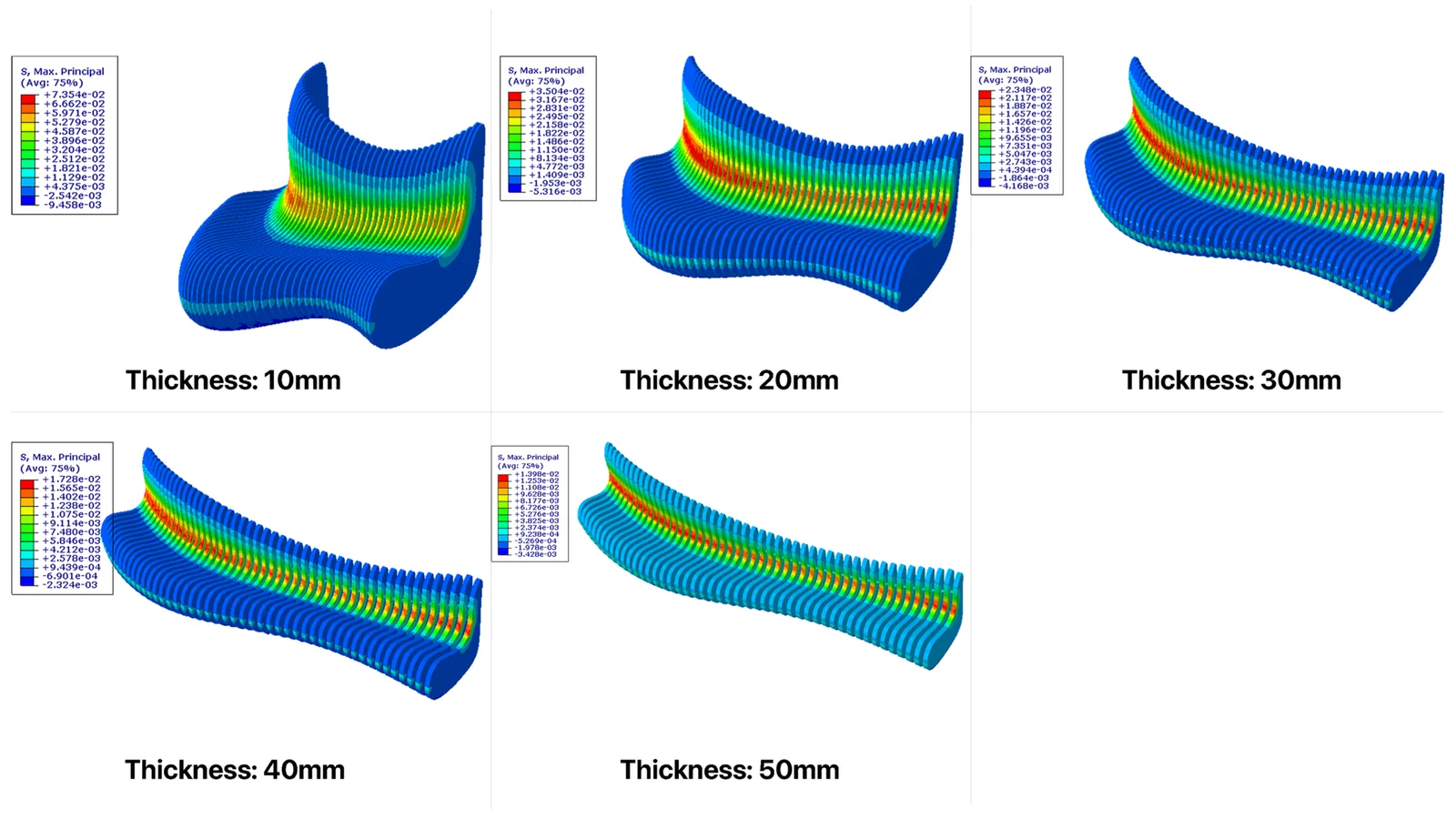
Simulations performed using NiTi construction materials for the bench, varying panels thickness.

The analysis was performed by varying the thickness of the parametric panels from 10 to 50 mm. As an example, in Figure 4 the resulting stress fields were reported for NiTi. Performance of stainless steel was omitted since its stress maps practically overlapped. Indeed, the stress distributions exhibit a consistent spatial pattern, with the highest stress concentrations occurring in the curved transition region between the seating surface and the backrest, which represents the principal load-transfer region of the geometry.

Increasing panel thickness progressively reduces the calculated stress levels under the same loading conditions. For NiTi, for example, the maximum critical stress decreases from approximately 0.073 MPa at 10 mm to 0.035 MPa at 20 mm, 0.024 MPa at 30 mm, 0.018 MPa at 40 mm, and 0.015 MPa at 50 mm, corresponding to an overall reduction of approximately 79% between the thinnest and thickest configurations. The progressively smaller reductions observed at higher thicknesses indicate diminishing benefits in terms of stress reduction alone. A comparable trend is observed for stainless steel, confirming that the geometrical effect of panel thickness dominates the stress trend under the adopted loading configuration.

Although the calculated stress magnitudes are low compared with the nominal strength of NiTi, this result is consistent with the adopted loading and geometrical configuration. The loads prescribed according to EN 1728:2012 [32] were implemented as uniformly distributed pressures over the relatively large seating and backrest surfaces and were further distributed through the 48-panel parametric geometry. Consequently, relatively low local stress levels are obtained under the considered service loading conditions. The purpose of the analysis is therefore not to approach the material strength limit, but to compare the stress response of alternative material and thickness configurations under identical loading and geometrical conditions.

Importantly, the present analysis is intentionally limited to stress-based comparison. Displacement was not investigated as a quantitative response variable and, therefore, no conclusions regarding displacement reduction or stiffness improvement are drawn from these simulations. Similarly, the term “optimal thickness” is not adopted, since no formal optimisation problem involving an objective function and explicit constraints was formulated. The investigated thicknesses should instead be regarded as a parametric design range used to evaluate the sensitivity of the stress response to geometrical variation.

Material-dependent differences would be expected to become more evident in a displacement-based comparison, since the different elastic moduli directly affect the deformation response even when similar stress–thickness trends are observed. Such a displacement-based comparison was not included in the present study and is therefore identified as a possible extension of the structural analysis rather than used to support the present conclusions.

Within this scope, the comparison between NiTi and stainless steel indicates that the introduction of the smart material does not produce a fundamentally different stress-distribution pattern under the considered passive loading conditions. This result is relevant to the objective of the present work: smart materials are not proposed as substitutes for conventional materials merely on the basis of passive structural performance. Rather, their potential value lies in integrating adaptive or responsive functionalities into conventional structural design when such functionalities provide measurable benefits capable of justifying their additional environmental and economic burdens. Therefore, the stress analysis should be interpreted as a structural compatibility assessment supporting the broader sustainability-driven comparison, rather than as evidence of superior mechanical performance of the smart material.

## 5. Conclusions

Results demonstrate that sustainability-driven material selection should be guided by the balance between functionality, sustainability, and feasibility rather than by absolute material performance. Traditional materials generally provide superior cost efficiency, lower environmental impact and proven durability, whereas smart materials offer adaptive functionalities that may justify their adoption only in specific engineering applications. In particular, metallic smart materials such as NiTi and Magnetic Shape Memory Alloys remain limited by their high economic and environmental costs, while Shape Memory Polymers represent a more balanced alternative, combining adaptability with moderate sustainability performance. Within the investigated case study, conventional materials generally showed more balanced sustainability profiles, benefiting from established production chains, lower costs, mature recycling routes, and broader social acceptance. Smart materials, conversely, introduce adaptive and responsive functionalities that may justify their higher environmental and economic burdens when these functionalities provide a measurable advantage for the intended application. Among the smart materials considered, Shape Memory Polymers showed a more balanced sustainability profile than metallic smart materials, whereas NiTi and Magnetic Shape Memory Alloys were more strongly affected by economic and environmental burdens. The structural analysis further indicated that, under the passive loading conditions considered, NiTi and stainless steel exhibited comparable stress-distribution trends; therefore, the potential value of smart materials should not be inferred from superior passive structural performance, but rather from the additional functionalities that they can introduce into the design.

As a final comment, no material can be considered universally superior; instead, smart materials should be regarded as complementary solutions whose use must be justified by measurable functional and sustainability benefits. Consequently, material selection should focus on identifying the most appropriate material for a given engineering function rather than seeking a universally “best” solution. Overall, the proposed framework provides a quantitative and function-oriented decision-support approach for integrating conventional and smart materials into sustainable design, enabling their potential benefits to be evaluated against the environmental, economic, and social burdens associated with their implementation.

## Figures and Tables

**Table 1:** Definition of some types of indicators and their unit of measure.

Sustainability Dimension	Type of Indicator	Unit
environmental	CO_2_ emissions	kg CO_2_/kg
	embodied energy	MJ/kg
	water consumption	L/kg
	renewable resource content	%
	recyclability	%
	waste generation	kg_waste_/kg
economic	material cost	€/kg
	service life	years
	maintenance requirements	€/year
social	occupational safety	score
	job creation	score
	community benefits	score
	social impact	score

**Table 2:** Definition of elastic moduli, Poisson ratios and density of the materials compared in this study.

Material	Young’s Modulus, (GPa)	Poisson’s Ratio	Density, (kg/m^3^)
NiTi (shape memory alloys)	70	0.33	6450
magnetic SMA (Ni-Mn-Ga)	15	0.30	8000
Shape Memory Polymers (SMPs)	2.0	0.35	1100
stainless steels 304	193	0.29	8000
Oak wood	12	0.35	700
natural stone (granite)	50	0.25	2700
recycled polypropylene	1.5	0.42	900
concrete (precast reinforced/RC 40/50)	35	0.20	2500

**Table 3:** Quantitative sustainability indicators collected from literature for the investigated conventional and smart materials. M: mass; GWP: CO_2_ emissions, C: cost, WI: water intensity, D: durability, EE; embodied energy, YS: yield strength, R: recyclability, WG: waste generation, A: accidents, JC: job creation, CB: community benefits, SI: social impact.

Material	M, kg/Panel		GWP, kg/kg	C, €/kg	WI, L/kg	D (0 to 1)	EE, MJ/kg
NiTi (shape memory alloys)	32		13	300	30	0.8	260
magnetic SMA (Ni-Mn-Ga)	40		15	800	35	0.4	320
Shape Memory Polymers (SMPs)	5		2.4	40	65	0.4	37
stainless steels 304	38		3.05	3.5	6	0.6	90
Oak wood	3.5		0.26	2.5	500	0.6	10.4
natural stone (granite)	13		0.23	0.8	2	1	0.8
recycled polypropylene	4		0.33	1.5	12	0.4	29
concrete (precast reinforced/RC 40/50)	12		0.14	0.15	2.5	0.8	1.9
**material**	**YS, MPa**	**R, %**	**WG, kg/kg**	**A/10^6^ worked hours**	**JC**	**CB**	**SA**
NiTi (shape memory alloys)	560	30	0.2	0.017	2	3	2
magnetic SMA (Ni-Mn-Ga)	250	20	0.3	0.017	1	2	1
Shape Memory Polymers (SMPs)	25	30	0.08	0.01	2	3	2
stainless steels 304	215	95	0.1	0.017	4	3	5
Oak wood	40	70	0.05	0.05	5	5	5
natural stone (granite)	130	80	0.15	0.015	4	4	4
recycled polypropylene	30	100	0.06	0.016	4	5	4
concrete (precast reinforced/RC 40/50)	40	90	0.12	0.015	5	4	5

## Data Availability

The original contributions presented in this study are included in the article. Further inquiries can be directed to the corresponding author.
